# Surface Modification of Silicon Nanowire Based Field Effect Transistors with Stimuli Responsive Polymer Brushes for Biosensing Applications

**DOI:** 10.3390/mi11030274

**Published:** 2020-03-06

**Authors:** Stephanie Klinghammer, Sebastian Rauch, Sebastian Pregl, Petra Uhlmann, Larysa Baraban, Gianaurelio Cuniberti

**Affiliations:** 1 Institute for Materials Science and Max Bergmann Center of Biomaterials, TU Dresden, 01062 Dresden, Germany; stephanie.klinghammer@nano.tu-dresden.de (S.K.); sebastian.pregl@nano.tu-dresden.de (S.P.); gianaurelio.cuniberti@tu-dresden.de (G.C.); 2 Leibniz Institute für Polymerforschung Dresden e.V., 01069 Dresden, Germany; Rauch@ipfdd.de (S.R.); uhlmannp@ipfdd.de (P.U.); 3 Department of Chemistry, Hamilton Hall, University of Nebraska-Lincoln, 639 North 12th Street, Lincoln, NE 68588, USA; 4 Center for Advancing Electronics Dresden, TU Dresden, 01062 Dresden, Germany

**Keywords:** field effect transistor, polymer brushes, silicon nanowire, bio sensing, dual-gate, Schottky barrier, Saos-2 cells

## Abstract

We demonstrate the functionalization of silicon nanowire based field effect transistors (SiNW FETs) FETs with stimuli-responsive polymer brushes of poly(N-isopropylacrylamide) (PNIPAAM) and poly(acrylic acid) (PAA). Surface functionalization was confirmed by atomic force microscopy, contact angle measurements, and verified electrically using a silicon nanowire based field effect transistor sensor device. For thermo-responsive PNIPAAM, the physicochemical properties (i.e., a reversible phase transition, wettability) were induced by crossing the lower critical solution temperature (LCST) of about 32 °C. Taking advantage of this property, osteosarcomic SaoS-2 cells were cultured on PNIPAAM-modified sensors at temperatures above the LCST, and completely detached by simply cooling. Next, the weak polyelectrolyte PAA, that is sensitive towards alteration of pH and ionic strength, was used to cover the silicon nanowire based device. Here, the increase of pH will cause deprotonation of the present carboxylic (COOH) groups along the chains into negatively charged COO^−^ moieties that repel each other and cause swelling of the polymer. Our experimental results suggest that this functionalization enhances the pH sensitivity of the SiNW FETs. Specific receptor (bio-)molecules can be added to the polymer brushes by simple click chemistry so that functionality of the brush layer can be tuned optionally. We demonstrate at the proof-of concept-level that osteosarcomic Saos-2 cells can adhere to PNIPAAM-modified FETs, and cell signals could be recorded electrically. This study presents an applicable route for the modification of highly sensitive, versatile FETs that can be applied for detection of a variety of biological analytes.

## 1. Introduction

There is an unceasingly high demand to develop novel biosensor platforms due to their wide range of potential applications in the fields of biotechnology, medicine, chemical analysis, and environmental monitoring [1]. Thereby, it is required to analyze, qualify, and quantify effects and processes that happen at the interface between biological systems and (bio-)sensors, and latter to transduce these signals adequately. Electrochemical sensors are capable of transforming biological signals into electric ones while operating rapidly, with low detection limits, and are easy to integrate into microelectronic circuits [2,3,4,5,6]. In turn, field effect transistor devices can play a key role in the aforementioned applications (e.g., biosensing), as the majority of biomolecules and bioreactions involve charge and potential shifts that can be detected electrically [7,8,9]. Silicon nanowires (SiNWs) do not only provide excellent electrical but also superior optical properties, enabling their usage also as optical biosensors [10,11,12]. Furthermore, modern semiconducting manufacturing techniques offer rewards in terms of miniaturization, parallel sensing, and integration [13,14]. Here, among other systems [15,16,17], silicon nanowire structured field effect transistors (FETs) are suitable for detecting of a wide variety of biological entities (i.e., DNA, nucleic acids, proteins, viruses, and cells), each with supreme limit of detection [18,19,20,21,22,23,24]. 

One of the main challenges in the area of biodetection is not only immobilization of receptors or biological entities but also their full removal from the surface to facilitate reusability of the sensor platform. 

Within the field of surface modification materials, polymer brushes have earned great attraction. They allow not only for a chemically stable attachment and homogeneous surface coverage, but they also provide biocompatibility. Their water compatibility, wettability, protein resistance, and non-fouling properties offer a wide variability in biological applications. The aforementioned properties can be altered reversibly by recurring changes of simple physical and chemical parameters such as pH and temperature. Stimuli-responsive polymer brushes can easily adapt to changes of surrounding environments and, hence, represent highly responsive surfaces [25,26,27,28]. Among reconstructable surfaces made of polymeric materials, brushes possess the advantage to respond very quickly and are long-lasting on incoming events. In addition, there is no corrosion or degradation of the film, so a high reversibility of surface properties can be realized [29]. The viability, responsiveness, and sensitivity of polymer brushes make them perfect candidates for use in biosensors, drug delivery systems, diagnostics, tissue engineering, and optical systems [27].

A specific chemical function and structure is required to maintain biological functions [30], but chemical modification of brushes can be used to tailor the surface properties for protein and cell adhesion, especially for applications in tissue engineering, bio-separation processes, and biosensing [31,32]. 

In sensoric applications, stimuli-responsive polymer brushes offer effective transduction mechanisms and have been used for pH sensors [33], chemical gating [34], microgravimetrics [35], optical transductions [36], and as acoustic sensors [37]. In addition, the device performance can be improved by modifying the sensors with stimuli-responsive polymer brushes [29,38,39]. For instance, for a glucose sensor the detection limit was decreased from 20 μM down to 2.5 × 10^−3^ μM when functionalizing the electrodes with polymer interfaces [40]. Though, polymer brushes are advantageous for probing catalytic reactions, enzyme recognition, and small molecule detection [28,41,42,43]. Lately, approaches have been made to combine the excellent transducing capability of SiNW FETs and the benefits from versatile polymer functionalization [44,45,46]. For instance, the switching events of brushes could be detected with FETs [47,48], dielectric materials were coated to create non-fouling surfaces [49,50], and chemical reactions were performed and transduced with polymer brush-FET systems [51,52]. 

The object of this work is to combine the advantageous properties of polymer brushes as an intermediate layer between sensor surface and receptors with the well performing detection capability of SiNW-FETs. Both parts can be manufactured separately and custom oriented at low cost and with high reproducibility. The combination of both will improve the functionality of the whole system and offers a promising tool for development of a sensor platform.

In this study we investigated the effect of two stimuli-responsive polymer brushes towards the performance of bottom-up grown SiNW-based FETs with Schottky junctions. We particularly focused on the polyelectrolyte poly(acrylic acid) (PAA). The pH-dependent swelling of PAA behavior is widely studied and is known to bind high quantities of biomolecules [53,54,55]. Surface modification with a pH-sensitive polyelectrolyte brush (PAA) was found to increase the pH sensitivity of SiNW-based FET sensors. Furthermore, poly-N-isopropylacrylamide (PNIPAAM) was chosen to create a biocompatible surface [56]. PNIPAAM is a temperature-sensitive polymer brush, which undergoes a sharp phase transition at its lower critical solution temperature (LCST) of 32 °C [57]. By introduction of temperature-sensitive PNIPAAM to the sensor platform, the wettability of surface was varied, and it was possible to control the interaction of the sensor with cells by changing the environmental temperature.

## 2. Materials and Methods

### 2.1. Chemicals and Reagents

Na_2_HPO_4_, NaH_2_PO_4_, KCl, HCl, NaOH, and PBS tablets were purchased from Sigma Aldrich (St. Louis, MO, USA). PBS tablets were dissolved in 200 mL deionized water. Chloroform (CHCl_3_), tetrahydrofuran (THF), and absolute ethanol (EtOH) were bought from Merck KGaA (Darmstadt, Germany). The polymers monocarboxy terminated poly(N-isopropyl acrylamide) (PNIPAAM-COOH), poly(acrylic acid) (PAA), and the adhesion promoter poly(glycidyl methacrylate) (PGMA) were purchased and characterized from Polymer Source, Inc. (Dorval, QC, Canada). Here, PGMA with molecular weight (MW) of 17.500 g/mol was chosen, whereas PAA had a MW of 26.500 g/mol and PNIPAAM of 47.600 g/mol. The positive photoresist AZ5214e was obtained by MicroChemicals GmbH, (Ulm, Germany). Cell culture reagents such as McCoy’s 5A, FBS, trypsin, L-glutamine, pen/strep, and sterile water were obtained from Biochrome AG (Berlin, Germany). Phosphate buffer solutions (0.1 M; Na_2_HPO_4_/NaH_2_PO_4_) with pH ranges from 5.7 to 8.0 were prepared.

Saos-2 cells were donated by Prof. Wiesmann, TU Dresden, IfWW. 

### 2.2. Silicon Nanowire Field Effect Transistors (SiNW-FETs)

**Figure 1:**
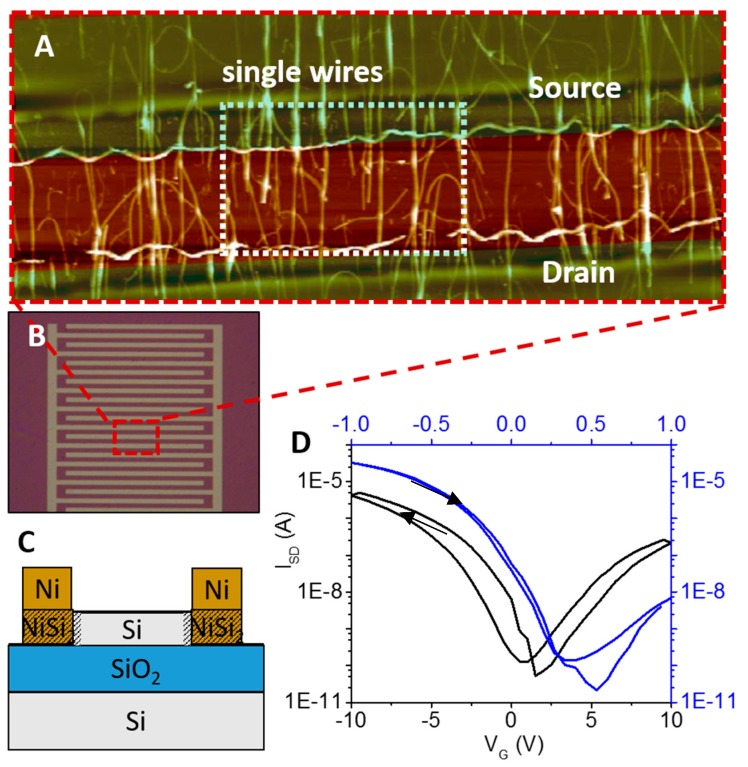
Representation of the sensor system. (**A**) AFM image of the silicon nanowire field effect transistors (FETs) with Schottky barriers. The electrodes offer multiple contact sites of the silico nanowires (SiNWs) to electrodes and create parallel arrays with numerous interconnections. (**B**) Interdigitated electrode design of the FETs. The red rectangle shows a magnification of the wire section. (**C**) Cross-section of the sensor. Nanowires are connected to electrodes via NiSi_2_ and serve as Schottky junctions. By the Schottky junctions the sensitivity of the sensor is enhanced. (**D**) Transfer characteristics of the SiNW FETs. The black line shows the behavior in dry conditions, whereas the blue line refers to characteristics in liquid conditions. The arrows indicate the scan direction of applied potential scan.

Sensors were produced as described previously by Pregl et al. [58]. Briefly, the silicon nanowires (SiNWs) were grown by chemical vapor deposition (CVD) on gold nanoparticles as seeds. Using this method, SiNWs from about 5–40 µm length and 20 nm diameter can be grown. In the next step, NWs were transferred mechanically by contact printing from the substrate to another chip substrate having a 200 nm thick SiO_2_ layer on top (see Figure 1). Nickel (Ni) electrodes were deposited via UV contact lithography, and subsequent heating to 500 °C supported the contacting between Si and Ni. Later, the chip was passivated with 20 nm aluminum oxide (Al_2_O_3_) via atomic layer deposition (ALD). The ALD process was performed in 150 cycles at 150 °C. Each cycle consisted of injection of trimethylaluminum for 0.35 s, followed by a purging step for 0.75 s. Subsequently, ozone was introduced and left for reaction for 15 s and purged for 10 s. The Al_2_O_3_ layer was locally etched via UV lithography (MJB 4 mask aligner, Süss MicroTec, Garching, Germany) using a 1 µm high layer of positive photoresist AZ5214e, post baked for hardening at 120 °C for 2 min. 

### 2.3. Electrical Measurements 

For electrical measurements, a digital source meter (Keithley 2602, Keithley Instruments, OH, USA) in combination with a probe station, consisting of two micromanipulators (Süss MicroTec, GArching, Germany) with tungsten needles, a microscope (Olympus, Tokyo, Japan), and a data acquisition system was employed. The experimental setup, including electrical circuit, is schematized in Figure S1A. Briefly, under ambient conditions, the source–drain current I_SD_ was recorded as a function of the gate voltage V_G_ that was swept from −10 to 10 V and back, while a fixed source–drain voltage V_SD_ = 25 mV was maintained. Data recording and analysis were performed with help of the software MATLAB© (R2012b, MathWorks, Natick, MA, USA). 

For temperature-dependent measurements, a home-made Peltier element, coated with a thin metal layer, was used to heat the sensors. 

### 2.4. Real-Time Measurementsof SiNW FETs for Biosensing Applications

The experimental setup, as mentioned in previous paragraphs, was improved for biosensing experiments. The samples were placed in a flow chamber, which contained a micro fluidic polydimethylsiloxane (PDMS, Dow Corning “Sylgard 184”) channel (2 × 15 × 0.5 mm). The fluid inside the microfluidic channel was driven by a syringe pump (Harvard Apparatus PHD 22/2000, Holliston, MA, USA), allowing for well-defined flow rates of 100 µL/min. In addition to the backgate, a reference electrode (Ag/AgCl, Microelectrodes Inc., USA) was introduced to the tubing system. Gate voltages were applied to the backgate and reference electrode of the system to minimize electrical noises. The experimental setup, including electrical circuit, is schematized in Figure S1B.

The applied gate voltage was reduced to a range from −1 to 1V when operating in liquid surroundings. For real-time measurements, a constant gate sweeping mode was realized, where V_G_ was swept continuously and I_SD_ was recorded. 

### 2.5. Preparation of Polymer Brushes

Prior to the chemical binding reaction, Si wafers as well as SiNW FETs were treated with 10 s of oxygen plasma (SPI SUPPLY PREP2, West Chester, PA, USA, 100 W, 0.2 mbar) in order to activate surface groups. PGMA (0.02 wt%) in CHCl_3_ was spin-coated onto the sensors at 2000 rpm for 10 s with an acceleration rate of 1000 rpm/min (Spin 150, SPS-Europe B.V.). Subsequent heating under vacuum at 100 °C for 20 min formed ether bonds between epoxy groups of the PGMA and OH groups of the silicon oxide. A schematic drawing of the reaction is shown in Figure S2A. 

For the preparation of PNIPAAM brushes, a 1wt% PNIPAAM solution in THF was spin-coated with the same parameters onto the PGMA layer and annealed at 150 °C for 15 h under vacuum. Non- bound PNIPAAM was removed by further extraction in THF.

For the preparation of PAA brushes, a 1 wt% solution of PAA in EtOH was spin-coated (2000 rpm for 10 s with an acceleration rate of 1000 rpm/min) onto the PGMA layer and annealed at 80 °C for 30 min under vacuum. Ungrafted PAA was removed by extraction in EtOH.

**Figure 2:**
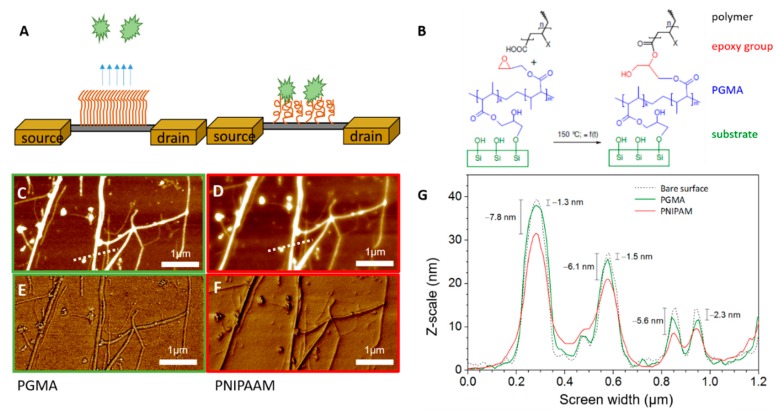
Functionalization of FETs with polymer brush PNIPAAM. (**A**) Schematic representation of polymer brushes immobilized on SiNWs. On the left side, the brushes stretch and, thus, repel biological entities. In contrast, when brushes are collapsed, biological moieties can adhere. Physical parameters like temperature, ionic strength, or pH can induce these conformational changes. (**B**) Chemical reaction for binding of polymer brushes to the linker layer PGMA via epoxy groups. (**C**) to (**F**) AFM images of Si nanowires after functionalization with linker layer PGMA and PNIPAAM respectively. Panels (**C**,**D**) depict the height images of the array upon modification. The dotted line represents the area where the height profile, which is shown in (**G**), was extracted from. In turn, panels E and F denote the phase images of the array. Here, homogeneous polymer layers were built up on the nanowires. (**G**) Height profile of SiNW FETs before (dotted line) and after immobilization of PGMA (green) and PNIPAAM (red).

Figure 2B shows the chemical reaction for both polymers. Since both polymers are COOH- terminated, one can assume the same reaction mechanisms for both polymers. The exact structural formulas for both polymers are given in Figure S2B,C. All samples were stored until further use in a dark, dry place at room temperature. 

### 2.6. Surface Characterization

Contact angle (CA) measurements were executed in static sessile drop mode with an OCA20 device (Dataphysics Instruments GmbH, Filderstadt, Germany). A 10µL volume of deionized water was placed on polymer-modified Si wafers at a flow rate of 1 µL/s. Five drops were applied on each sample. Temperature-dependent measurements were performed either at room temperature (23 °C) or at 40° C with tempering time of 15min, respectively.

Atomic force microscopy (AFM) was performed in tapping mode with Nanoscope IIIa, Dimension 3100 (Veeco, Plainview, NY, USA). Operation in air was used to control homogeneity and roughness of the surfaces after preparation and after pre-treatment at different pH. Tips of the type BSTap (Budget Sensors, Sofia, Bulgaria) with a resonance frequency of 300 kHz and a spring constant of 40 N/m were used. 

### 2.7. Cell Culture 

For cell culture experiments, the human osteosarcomic cell line SaoS-2 was used. All cell culture experiments were carried out in aseptic conditions. Prior to use, all solutions were sterile filtrated and autoclaved. The polymer and sensor samples used were sterilized with EtOH. SaoS-2 cells were cultured in McCoy’s 5A medium, which had been supplemented with 15% fetal bovine serum (FBS), 1% penicillin/streptomycin, and 1% L-glutamine. Detailed protocols for cell culture and sub-culturing are summarized in supplementary information. 

Sterile samples were placed in a 6-well plate (polystyrene, Nunc, Rochester, NY, USA), and cells at a concentration of 10^4^ cells/mL were seeded onto the samples and incubated for 24 h. For temperature-dependent experiments, samples were incubated at 25 °C, 5% CO_2_, and humidified atmosphere for times of interest. Prior to imaging, cell culture medium (CCM) change was executed to remove non-adherent cells. For pH-dependent experiments, acidic CCM was used, and pH was decreased slowly with 0.1 mol/L HCl until the favored pH was reached. 

## 3. Results

### 3.1. Sensor Fabrication and Characterization

Silicon nanowire-based sensors with Schottky junctions were fabricated as described previously [58]. The undoped silicon nanowires were grown via CVD using gold NP seeds and subsequently aligned on the sensor substrate to achieve a parallel array of nanowires [59,60]. This approach resulted in FETs with numerous bottom-up grown SINWs with about 5–40 µm length and 20 nm diameter. Via alignment of them, multiple interconnects between electrodes and wires were realized. In Figure 1C, a cross-section of the sensor can be seen. The interdigitated assembly of electrodes, as presented in Figure 1B, enables multiple interconnects of densely packed nanowires as shown in the AFM image of panel 1A. By introducing Schottky barriers to the sensors, a highly sensitive, rapid, and reliable sensor platform was generated [61]. Subsequent passivation with Al_2_O_3_ facilitated measurements even under harsh liquid conditions. Figure 1D shows the transfer characteristics of the devices in ambient conditions (black line) and liquid conditions (blue line). In both cases, a potential sweep from negative to positive voltages and back was applied. The sensors showed an on/off ratio of about 10^5^ A and a slightly hysteretic behavior due to the presence of dielectric oxide layers on the silicon surface. The oxide layer contained surface hydroxyl groups that will trap charges during measurement and will reflect as hysteresis. Schottky barrier based devices showed a sensitivity of −550 mV/dec in the subthreshold regime under dry conditions, whereas under liquid conditions the slope increased ~10 times to −5754 mVdec. In general, it has to be said that the slope of the characteristic was steeper under aqueous than in ambient conditions due to coupling effects between back and liquid gates. Moreover, only the most sensitive parts of the transfer characteristics were used for analysis. Here, the subthreshold regime was considered to be the most delicate one [62]. Due to their small dimensions, NWs are very sensitive to surface effects. The targeted aligned Al_2_O_3_ layer as well as the native SiO_2_ oxide forming on the side walls may influence the transfer characteristic of the wires substantially [63,64].

### 3.2. Sensor Functionalization with Stimuli-Responsive Polymers

Two different stimuli-responsive polymers were used to tailor the interfacial surface properties of silicon nanowires. By stimulating them with appropriate parameters, the polymers can switch their conformation and thus change their adhesion properties for biomolecules. The schematic principle is demonstrated in Figure 2A. The temperature-sensitive polymer poly(N-isopropylacrylamide) (PNIPAAM) and polyacrylic acid (PAA), which is sensitive to pH and ionic strength changes, were used. Both polymers were put onto surface via “grafting to” approach, where a polymer solution is allowed to react with surface that has been functionalized previously with a suitable coupling agent. A thin poly(glycidyl methacrylate) (PGMA) layer acted as those layers. The schematic chemical reaction for binding both polymers to PGMA is given in Figure 2B. 

The success of the functionalization was observed by AFM in tapping mode. Additionally, phase images were taken to confirm material composition on the surface. Here, roughness of the samples was only determined from planar surfaces and extracted from height images. Corresponding images from Al_2_O_3_-treated wafers are listed in Figure S3. The roughness (R) of an Al_2_O_3_-passivated surface was 0.38 nm on average, and the roughness decreased to 0.37 nm when PGMA was present on the surface. The functionalization with PNIPAAM resulted in a surface roughness of 0.37 nm and, thus, was found to be similar with the R of pure PGMA linker layer. In contrast, the roughness increased to 0.62 nm when PAA had been adsorbed. For PGMA as a linker layer, a homogenous coverage and, hence, an evenly structured surface can be seen in Figure S3, while for PNIPAAM a cloud-like structure and sticky surface could be observed. For PAA, AFM height images of the surfaces showed homogenously distributed structures with noticeable differences in height, leading to a comparable rough surface. As equally charged polymers aggregate close to each other, a higher roughness and local clusters are expected.

Exemplary AFM images before and after functionalization of the SiNW FETs with PGMA and PNIPAAM are depicted in Figure 2C,F. Here, panels C and E demonstrate the height and phase images of PGMA-modified surfaces, whereas panels D and F refer to PNIPAAM-modified sensors. Dense and homogeneously distributed polymer layers were created. Suitable images for PAA-functionalized brushes can be found in the SI, Figure S4. The height profile presented in Figure 2, panel G shows the height increased with each reaction step. Here, the height of the polymer on the wires changed from 1.3 up to 2.3 nm after PGMA adsorption. In contrast, when PNIPAAM was added, the height increased from 5.6 up to 7.8 nm. For PAA, height differences of about 1.4 to 2.5 nm occurred (data shown in Figure S4B). Parallel performed ellipsometry showed a PGMA thickness of 2.4 nm (± 0.1), a PNIPAAM thickness of 14.7 nm (± 0.1), and an average thickness of PAA of 5.2nm (± 0.7). By analyzing AFM images of similar wires after every treatment one can see that for both brush systems only half of the originally prepared layer remained at the wire surfaces. This is addressed by the fact that the viscose polymer solutions will be spun off the small surface area of the wires during preparation, and the majority of the solution will be deposited on the interspaces between the wires rather than on top of them. Since AFM height profiles were leveled to these interspaces, an overall decrease in thickness of the layer appeared. The difference between measured thickness with ellipsometry and those measured in AFM images will reflect the actual height of brushes on the nanowires. 

### 3.3. Phase Transition of Polymer Brushes

**Figure 3:**
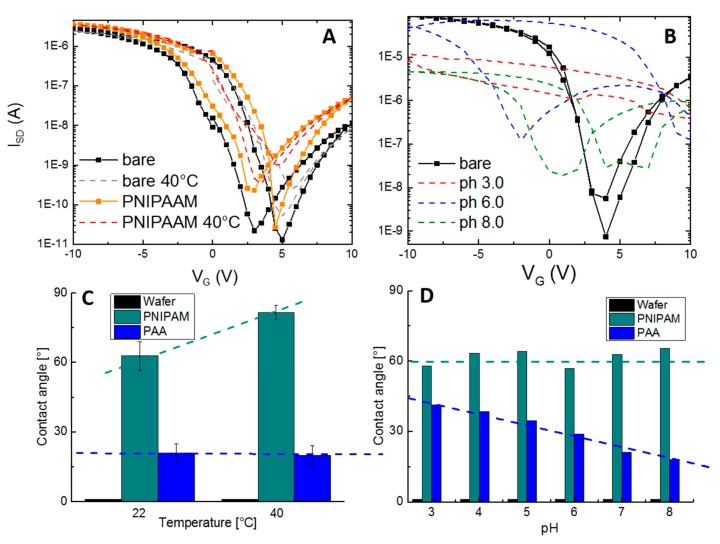
(**A**) Transfer characteristics of SiNW FETs with and without functionalization with PNIPAAM. Sensor response upon temperature increase above the lower critical solution temperature (LCST) is shown. There is no significant change in behavior from PNIPAAM-coated FETs compared to untreated devices, and brushes switch only under liquid conditions. (**B**) Transfer characteristics for unmodified and PAA-functionalized sensors upon immersion in three different pH solutions. With increasing pH, the chains of the brushes repel more to each other, leading to a growing gating behavior of the FETs. (**C**) Contact angles (CAs) of both brush systems upon temperature increase to 40 °C. For PNIPAAM, hydrophobicity increases when T>LCST. (**D**) Contact angles of both brush systems upon change of surrounding pH. Static CA declines linearly for PAA-modified surfaces when pH is raised due to phase transition.

Swelling, or rather phase transition, of polymer brushes can only take place under liquid conditions. The static, sessile drop contact angle (CA) measurements were executed to follow the transition event of each polymer brush. CA measurements were performed in dry state before and after treatment at different pH solutions as well as adjustment of temperature. Simultaneously, functionalized SiNW FETs were exposed to the same parameters, and transfer characteristics were recorded in ambient conditions too. Figure 3A,B represents the transfer characteristics of SiNW FETs modified with polymer brushes and upon stimulation with their suiting physical parameters (i.e., temperature and pH). Figure 3C,D characterizes the contact angles of both brushes upon exposure to mentioned physical parameters. 

The alteration of temperature influences only systems that have been functionalized with PNIPAAM. Here, increasing the temperature to 37 °C, and thus above the LCST of 32 °C, is expected to lead to a 5-fold decrease of thickness of the brushes [65]. Accordingly, as summarized in Figure 3C, the CA enlarged from 62.8° to 81.6°. A higher hydrophobicity is an effect of increasing interactions between the polymer chains. As depicted in Figure 3A, immobilization of PNIPAAM onto the SiNW FETs resulted in a slight right shift of the transfer characteristics, which was caused by a slightly negative net charge of the PNIPAAM chains. The subsequent increase of T resulted in further right shift of the FETs for both cases, bare FETs, as well as PNIPAAM-functionalized ones. As the (de-)swelling of PNIPAAM is driven by hydrophobic interactions and hydrogen bonds, and not by any exchange of charges, the shape and quality of transfer characteristics remained stable during temperature variations. Figure S2D summarizes the phase transition based on hydrogen bonds. For PAA-modified sensors, as expected, no temperature dependence was found. 

When the pH increased from 3.0 to 8.0, the change of this parameter caused an extension of the thickness of PAA about 2.5 times [65]. Therefore, as displayed in Figure 3D, the CA decreased almost linearly from 41.3° at pH 3 to 17.8° at pH 8. Parallel transfer characteristics of PAA-treated FETs are shown in Figure 3B. The incubation of the sensors at pH 3 resulted in a constant ON current state, whereas a further increase of the pH to 6 and 8 caused a gating effect of the FETs. The rather high hysteresis of the FETs and the impaired on/off ratios were caused by the numerous charges being present in the structure of the PAA chains. Figure S2E summarizes the structure of PAA and its charges in stimulated and non-stimulated conditions. At pH 3, the PAA was a swollen, closely packed system with carboxylic groups acting as charge carriers preventing the FETs from gating. In contrast, raising the pH to 8.0 loosened up the brush layer and presented charges that are expected to contribute to a gating effect of the FETs. The appearance of a hysteresis is dependent on water influence (e.g., humidity) or presence of electrolytes close to SiNWs. The traps were considered to be close to the surface of the silicon oxide, and adsorbed water molecules were expected to decrease the bonds in the first oxide layers. Thus, the trapping process was influenced since charges can be injected into this hydration layer, and transfer characteristics were influenced. It has to be noted that, prior to incubation to different pH solutions, the transfer characteristics of PAA-treated FETs remained close to its initial shape as the polymer is considered to be “dry”; thus, none of the described charge transfer effects will be affected. For PNIPAAM-modified FETs, no pH dependence could be investigated at any point. 

### 3.4. Real-Time Measurements of Stimuli-Responsive Polymer-Modifed SiNW-FETs 

Real-time measurements were performed to investigate the effect of temperature and pH increase towards pure and polymer-modified SiNW-FETs. As stimuli-responsive polymers undergo phase transitions only in liquid environments, FETs were placed in a microfluidic chamber allowing a constant exposure to the liquid. A detailed setup, including the electrical circuit of the system, can be found in Figure S1C,D. To evaluate the kinetics of phase transition, signals were recorded in real-time. A constant sweep mode was established, where the gate voltage swept continuously, and source drain currents were recorded simultaneously. As all information of electrical properties is recorded constantly, this method is regarded to enable quantitative measurements in an optimal regime, hence allowing for the latter extraction of threshold voltages in the most sensitive area of the FET. By this, a higher sensitivity of the system is expected [66].

**Figure 4:**
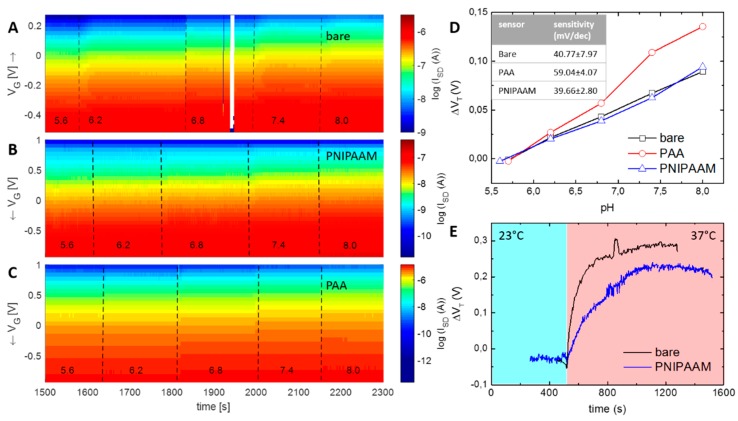
(**A**) Real-time response of the unmodified sensor upon exposure to different pH. (**B**) Real-time response of PNIPAAM-modified SiNW FET upon exposure to different pH. (**C**) Real-time response of the PAA-modified sensor upon exposure to different pH. Source–drain currents are measured with a continuous gate sweep measurement and are displayed as a function of gate voltage and time. (**D**) Summary of pH sensitivity for the differently functionalized sensors (data extracted from panel A to C). The pH-sensitive PAA brush enhances the sensitivity of the system slightly, whereas with PNIPAAM no significant difference to unmodified sensors could be seen. The inset summarizes data of three independent experiments. (**E**) Temperature sensitivity of bare and of PNIPAAM-treated sensors. Dynamics appear slightly slower for PNIPAAM-functionalized FETs.

The majority of experiments were executed as pH-sensing experiments. The influence of the pH changed the surface potential of the FETs and, thus, is the physical basis of the sensitivity of SiNW-based devices. Here, phosphate buffers ranging from 5.6 up to 8.0 were guided over FETs unremittingly. Figure 4 summarizes the FET response towards different pH solutions of untreated (panel A), PNIPAAM-modified (panel B), and PAA-modified (panel C) sensors in real time. Source–drain currents (I_SD_) for different pH values were depicted as a function of gate voltage (V_G_) and time. At a fixed V_G_, an increasing pH will reflect as an increase in the source drain current I_SD_. Extracted from these data, Figure 4D shows the sensitivity of the differently treated sensors. Unmodified and PNIPAAM-modified FETs showed sensitivities of ~40 mV/dec, whereas PAA-modified sensors showed an enhanced sensitivity of ~59 mV/dec, which is in turn close to the Nernst limit at 59.5 mV/dec. It is expected that the ion-sensitive polymer PAA acts as a magnifier for free ions due to the presence of numerous carboxyl groups. The counter ions will contribute to the interaction of surface potential of SiNW FETs. With increasing pH values, dissociation of the carboxyl-groups proceeded, and more negative charges were introduced into the system, explaining the higher sensitivity for PAA-modified FETs. Dong et al. [67] determined that there is a dissociation gradient inside the PAA brushes. Increased charge regions are present at higher distances from the substrate surface being caused by the minimization of the system’s free energy [68], an inhomogeneous polymer volume fraction, and the formation of a double layer at the brush–solution interface [69]. In addition, the presence of PAA can be considered as another capacitive layer occurring on the surface. Regarding the polymer film as an additional dielectric layer, an increase of the equivalent oxide thickness (EOT) is expected. Assuming that most of the brush film consists of water with permittivity of ɛ_R_ = 80 at 20 °C and partly of polymers with ɛ_R_ = 3 [70], the fraction of polymer in calculation of EOS can be neglected. Taking the water as further increase of oxide thickness, this would lead to a higher sensitivity factor and thus to higher sensitivities compared to non-functionalized surfaces. In contrast, more inert surface groups, as present inside the PNIPAAM-structure, do not interact with the charge transfer principles during gating and thus remain comparable to those of unmodified sensors. 

Phase transition of PNIPAAM was stimulated by temperature changes. Figure 4E shows the electrical response of unmodified and PNIPAAM-modified sensors upon heating from 23 °C to 37 °C. The actual heating reflects within the signal by an increase of V_T_ of ~250 mV at a temperature difference of 15 K. PNIPAAM sensors reacted slightly slower to the temperature increase than untreated ones. As the polymers build up an additional layer on the wires, more mass has to be heated until full temperature balance is achieved. As a consequence, the increase of ΔV_T_, which is considered to equal the heating dynamics, appears slower until equilibrium is attained. After full temperature compensation, no differences between untreated and PNIPAAM modified FETs were detectable. Hence, the actual collapsing could not be confirmed electrically. This observation is based on the fact that the phase transition of PNIPAAM occurs within milliseconds [71] and thus is limited by the heating dynamics of the sensors. 

### 3.5. Cell Adsorption on Polymer Brushes

As a last step, exemplary selected cell adhesion of osteosarcomic Saos-2 cells onto corresponding surfaces was investigated. Cells were incubated on glass reference substrates imitating bare FETs, PNIPAAM, and PAA-modified ones. After 24 h of cell culture on the surfaces, cell adhesion was determined via optical microscopy, and stimuli conditions were altered. The temperature decreased from 37 °C to 23 °C to cause stretching of PNIPAAM brushes and to force detachment of cells. 

**Figure 5:**
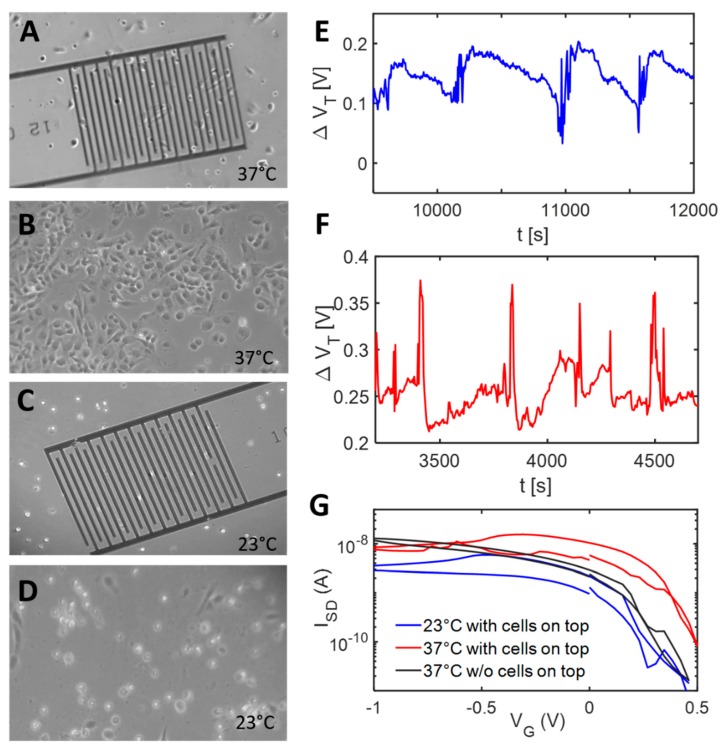
(**A**,**B**) Microscopy images of Saos-2 cells incubated at 37 °C on PNIPAAM-functionalized sensors (**A**) and planar reference substrates (**B**). A strong cell adhesion can be seen in both cases, indicating viable cells. (**C**,**D**) Microscopy images of Saos-2 cells at 23 °C on PNIPAAM-functionalized sensors (**C**) and planar reference substrates (**D**). Lowering the temperature to 23 °C results in detachment of the cells, as mainly spherical residues can be seen. E) - F) Real-time sensor response of PNIPAAM-coated FET at 23 °C (**E**) and 37 °C (**F**). In both cases, a periodically appearing signal can be seen when cells do come in contact with sensor surface. (**G**) Transfer characteristics of sensors with and without cells. (Data extracted from time points of panels E and F, where spikes are present.) If cells are present, the capacitive part of the curve is more pronounced. In contrast, there is only small hysteresis when no cells are present (black curve).

Figure 5A,B shows the cell adhesion on PNIPAAM-modified sensors and unstructured surfaces at 37 °C. The majority of the cells adhered thoroughly and evenly spread over the surfaces. If there were electrodes under the cells, slightly less cells adhered. The decrease in cell quantity is considered to be caused by free metal ions penetrating into the solution and by interferences originating from the structure of the surface. In Figure 5C,D, the temperature decreased to 23 °C, and cell detachment took place, as there were only spherical cell morphologies observable. However, further re-increase of the temperature to 37 °C resulted in a re-adhesion of the cells. Furthermore, PAA-treated as well as unmodified surfaces were investigated, and suitable images are presented in Figure S5. For both control surfaces, a similar cell adhesion was observable, which was not influenced by temperature changes. Only small detachment was detected at control surfaces, noticeable by slightly spherical-shaped cells on the surfaces. 

In contrast, the decrease of the pH resulted in enhanced apoptosis rates of the cells, independent from surface characteristics (data not shown). It is known that cell death occurs after brief exposure to acidic conditions [72,73,74,75]. Due to the aforementioned reasons, electrical detection of cells was only performed on PNIPAAM modified FETs.

### 3.6. Electrical Detection of Saos-2 Cells

For electrical measurements, cells were resuspended in pure PBS, as the complexity of the cell culture medium would interfere negatively with the FETs. On the one hand, enhanced degradation of the electrodes will take place. On the other hand, numerous proteins and other (bio-)molecules will adsorb to the sensor surface leading to misleading signals of the FETs. 

SaoS-2 cells were placed on the sensor at a density of 10^5^ cells/mL, and cellular responses were investigated at 37 °C and 23 °C. Figure 5E,F shows the real-time response of PNIPAAM-modified FETs towards cell exposure. For both temperatures, periodically appearing signals can be detected indicating cellular activity on or nearby the sensor surface. At 37 °C, the occurring spikes appear upwards, whereas at low temperature they are directed downwards. It is expected that the positions of the signals correspond to the presence of a cell on the surface, and transfer characteristics shown in Figure 5G are extracted from those time points. When there was a cell present on the sensor, an enhanced hysteresis was revealed. It is expected that the hysteresis originates from changes in surface capacitance induced by the cells, as Si NW/SiO_2_ traps are constant in our system. It is noteworthy that directly after application of the electrical field, no cell adhesion could be observed anymore. The electrical field will not only modify cell morphologies and cellular structures, but also properties related to cell adhesion [74,75]. Thus, introducing cells into the system led to short periodic signals, which are expected to be either electrical signals originating from the cells (cells tend to respond toward external stimuli via electrical signals) or converging cells, which are endeavored to adhere but are repelled by the electrical field.

## 4. Summary

We performed functionalization of Schottky junction based SiNW FETs with two stimuli-responsive polymer brush systems successfully. The thermo-responsive polymer PNIPAAM and the pH- and ionic strength-dependent polymer PAA were investigated. Both polymers underwent reversible conformational changes when being exposed to their external stimuli, which could facilitate or hinder adhesion of biomolecules (e.g., proteins, cells) respectively. The reversible swelling and collapse of the brushes were confirmed by AFM and static contact angle measurements. In addition, the changes of the chemical and physical properties were monitored with modified FET devices. 

As both polymer coatings are considered to be biocompatible, the suitability of the system as a biosensor is encouraged. Particularly, we focused on the temperature-dependent, reversible adhesion of cells on PNIPAAM-modified sensors and its electrical detection. A reversible cell adhesion and detachments could be proven while the electrical signals remained unchanged upon modification. We were able to detect signals originating from osteosarcomic Saos-2 cells. In addition, functionalization with the weak polyelectrolyte PAA enhanced the pH sensitivity of the FETs. The adhesion of biomolecules to PAA brushes via electrostatic interactions can be easily adjusted by pH. 

In conclusion, the chosen polymer brushes provide a good complement to SiNW-based FETs, as the actual FET response is not changed dramatically, but a biocompatible surface is created. Furthermore, a high reversibility of the swelling properties promotes high tunability of both systems and suits the application of brushes as sensors. The usage of a mixed polymer brush system consisting of PNIPAAM and PAA would result in a system with new properties and would open up new possibilities for application.

## Supplementary Materials

The following are available online at https://www.mdpi.com/2072-666X/11/3/274/s1. Figure S1: The experimental setup, including electrical circuit, Figure S2: Schematic drawings of the reaction, Figure S3: AFM height and phase images of differently modified glass slides during modification with polymer brushes, Figure S4: (A) AFM height and phase images in two magnifications of SiNW FETs during modification with polymer PGMA and later PAA. White rectangles show area, where magnifications were taken from. White line indicates the position where height profile was extracted. (B) Height profile of SiNW FETs during functionalization with PGMA and PAA, Figure S5: Incubation of cells on PNIPAAM, PAA and glass surfaces.
