# Supplementary material for: Surface Modification of Silicon Nanowire Based Field Effect Transistors with Stimuli Responsive Polymer Brushes for Biosensing Applications
Source: Micromachines (Basel). 2020 Mar 6;11(3):274. doi: 10.3390/mi11030274 (PMC7143225; doi:10.3390/mi11030274)
Supplement: Supplementary file 1 [file micromachines-11-00274-s001.pdf]

micromachines

Supplementary Materials: Surface Modification of Silicon Nanowire Based Field Effect Transistors with Stimuli Responsive Polymer Brushes for Biosensing Applications

Stephanie Klinghammer , Sebastian Rauch , Sebastian Pregl , Petra Uhlmann , Larysa Baraban and Gianaurelio Cuniberti

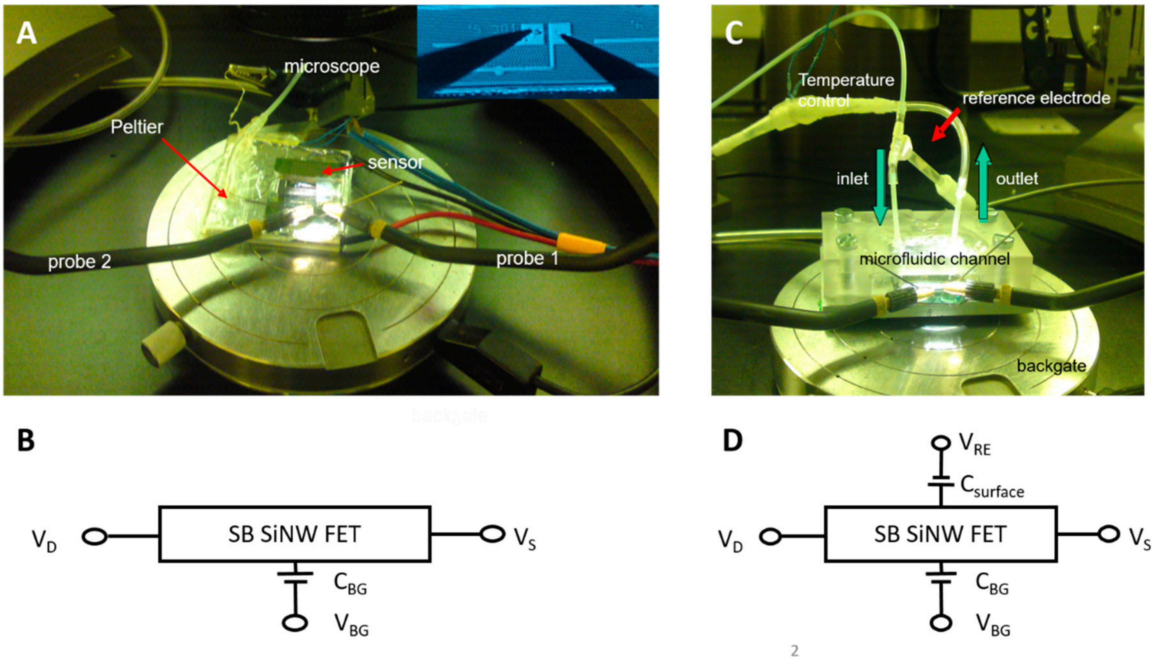

Figure S1. The experimental setup, including electrical circuit (**A**) Experimental setup in ambient condition with Peltier-element driven temperature control. Gate voltage to Si NW FETs is applied via back-gate (BG). (**B**) Schematic electrical circuit of back-gated SiNW FET. (**C**) Experimental setup for liquid measurements. In a dual-gate setup, the back-gate and liquid gate are connected to SiNW FETs. The FET is embedded in a microfluidic channel system allowing for analyte flow. (**D**) Schematic electrical circuit of dual gated SiNW FET.

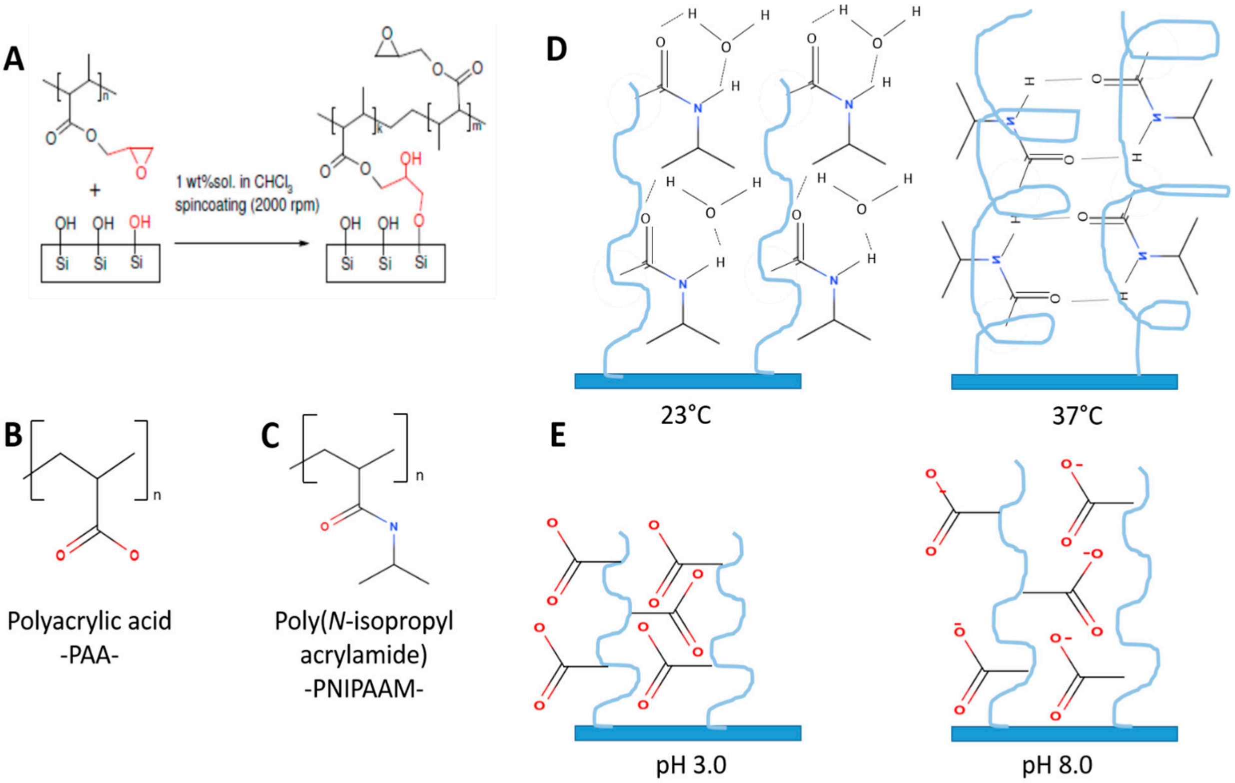

Figure S2. Schematic drawings of the reaction (**A**) Chemical reaction of FETs with initiator molecule PGMA. (B) structural formula of PAA (**C**) Structural formula of PNIPAAM (**D**) Phase transition of PNIPAAM. At 37 °C, polymer brushes are collapsed and biolmolecules can adhere. (**E**) Phase transition of PAA. At high pH values, carboxyl groups are deprotonated and chains repel from each other due to negative charges.

Sample	Height image	Phase image
Al_2O_3; x=3 µm; z= 4 nm/4°	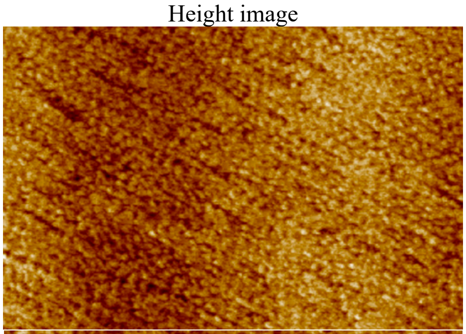	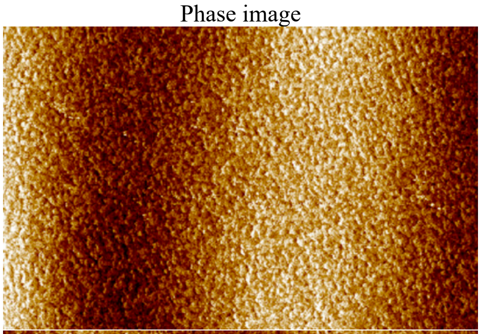
PGMA; x=3 µm; z=4 nm/4°	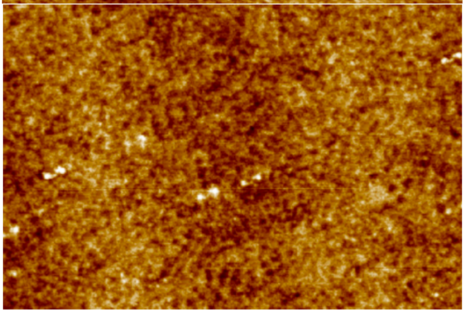	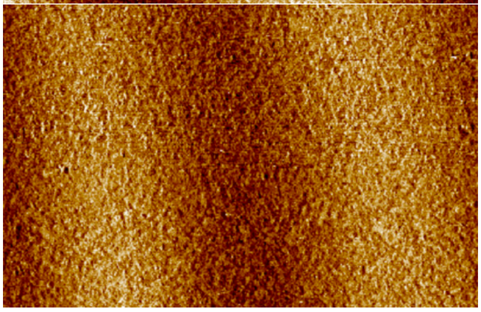

PNIPAM; x=3 µm; z= 4 nm/4°

PAA; x=3 µm; z= 4 nm/4°

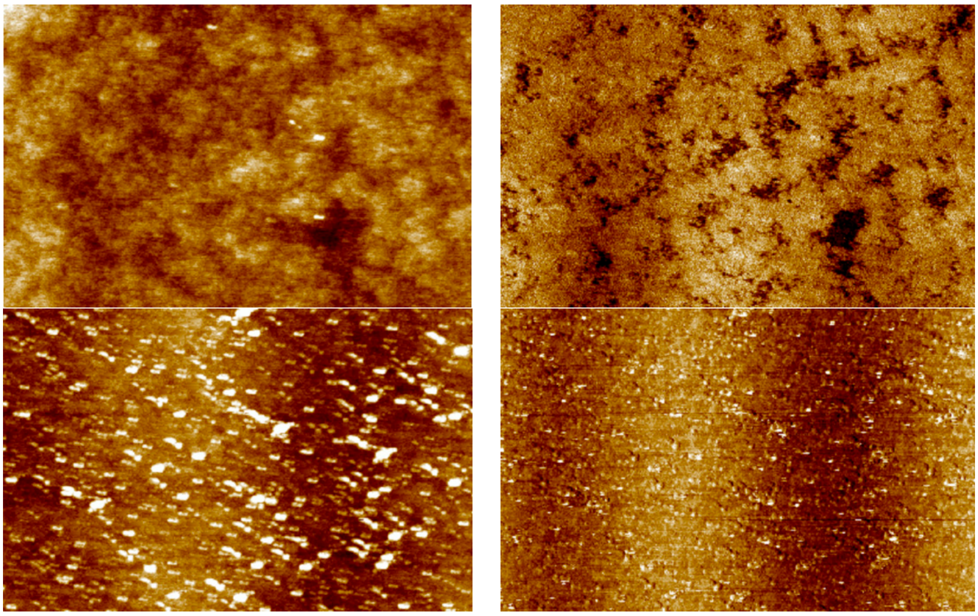

Figure S3. AFM height and phase images of differently modified glass slides during modification with polymer brushes. Functionalization with PNIPAAM leads to cloud-like structures whereas the exposure to PAA lead to evenly rough surfaces.

A

Height image | Phase image

PGMA; 10 µm, z: 80 nm/16°

PGMA; magnification from a) (3 µm, z: 80 nm/16°)

PAA; 10 µm, z: 80 nm/16°

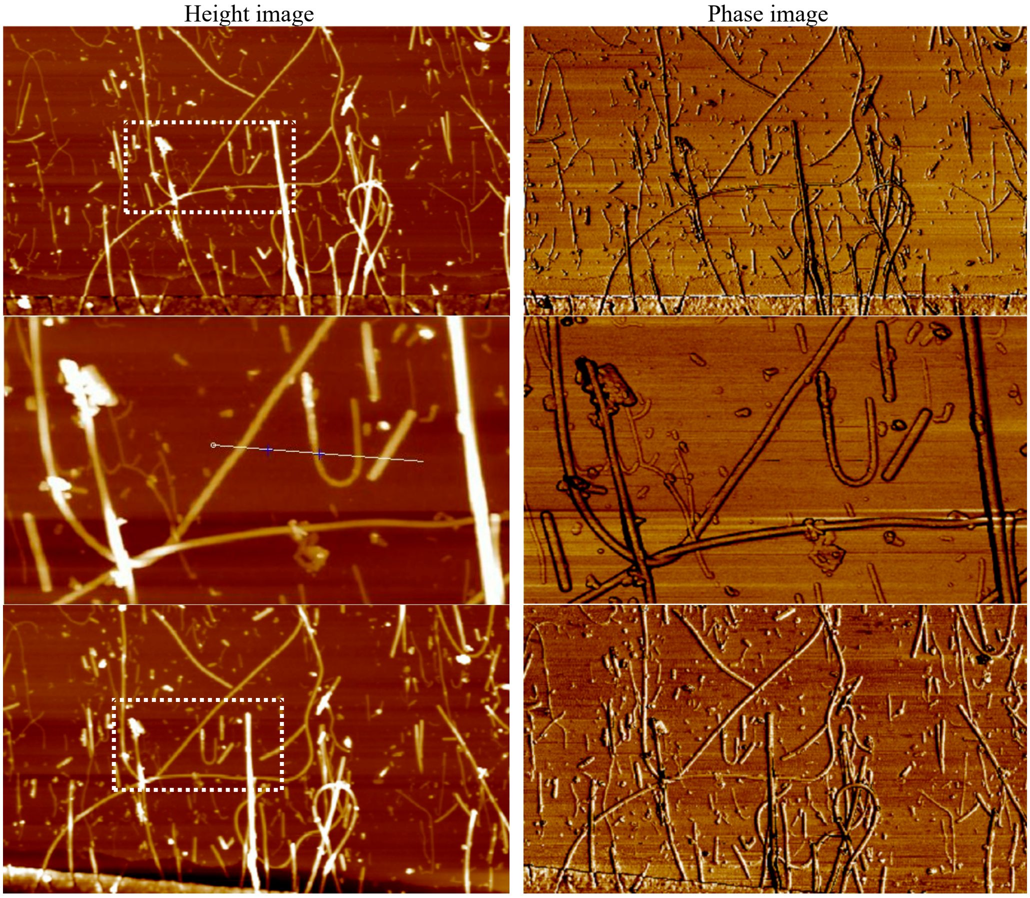

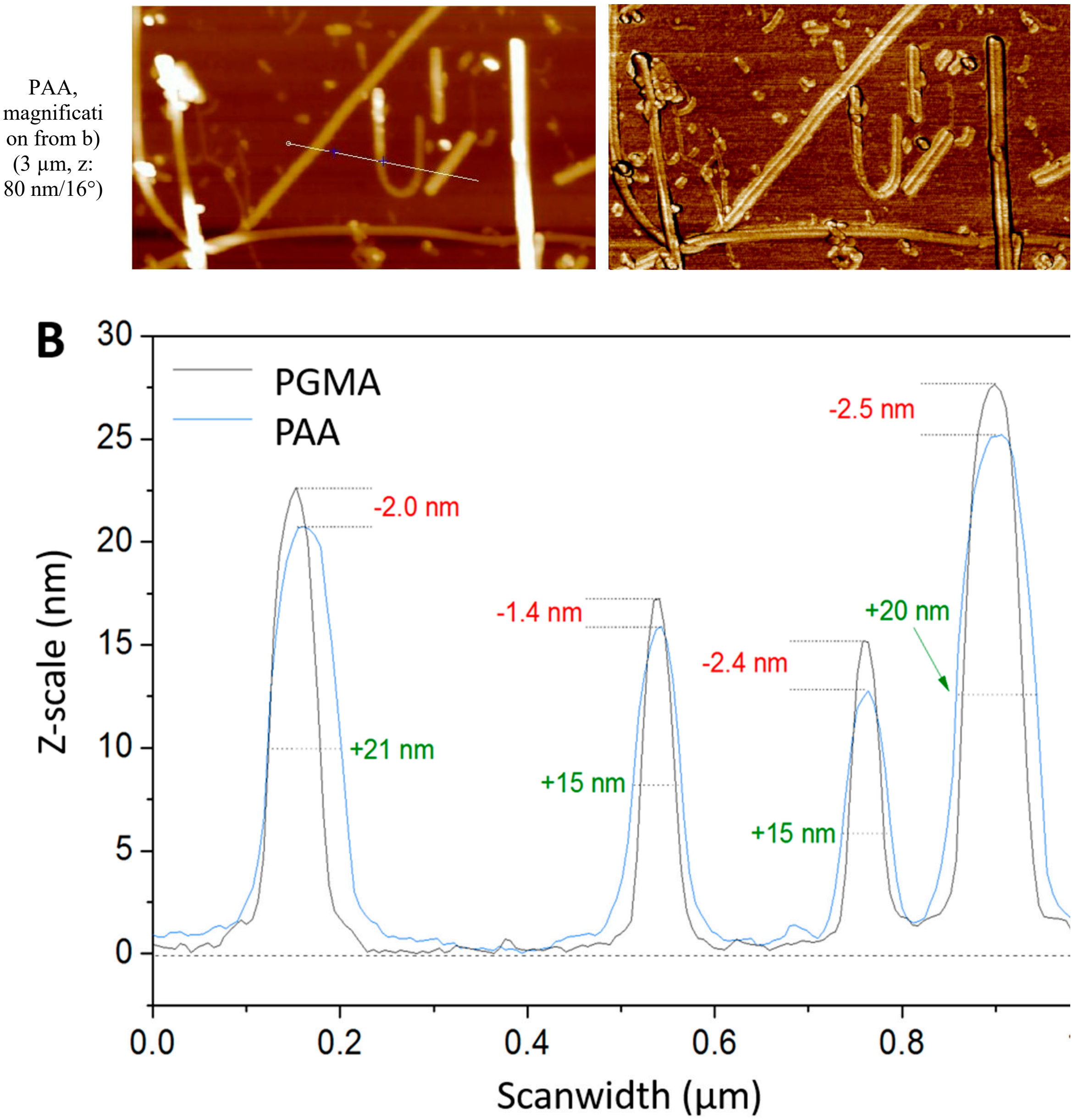

Figure S4. (**A**) AFM height and phase images in two magnifications of SiNW FETs during modification with polymer PGMA and later PAA. White rectangles show area, where magnifications were taken from. White line indicates the position where height profile was extracted. (**B**) Height profile of SiNW FETs during functionalization with PGMA and PAA.

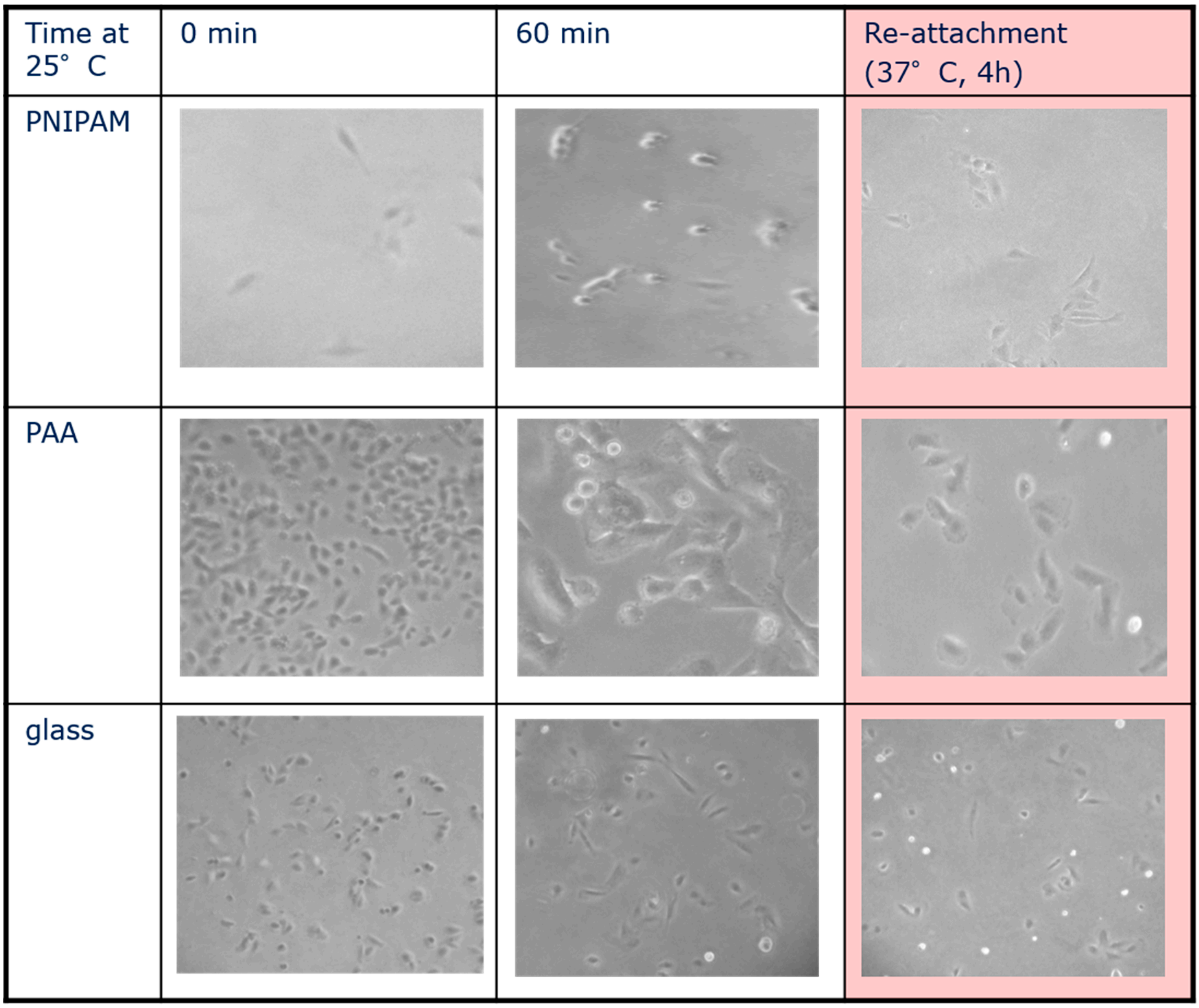

Figure S5. Incubation of cells on PNIPAAM, PAA and glass surfaces. The cells were incubated at 37 °C (left column). Subsequent lowering of the temperature to 25 °C leads to detachment of cells on PNIPAAM surfaces, whereas on PAA and glass the cells remain adsorbed (center column). Re-heating of the substrates to 37 °C leads to readhesion of the cells.

Additional Protocols for Cell Culturing

Culturing SaoS- 2, starting from subculture 16

Cells were stored at −196°C in liquid nitrogen in so called cryo- tubes and defrosted at 37°C in a water bath. Subsequently the cell suspension (1106 cells/ml) was added drop wise to 10ml room tempered medium, centrifuged at 1500 rpm for 3 min, resuspended in 30 mL CCM, 37 °C and seeded into a 175 cm^2 flask. Afterwards cells were maintained in a humidified atmosphere at 37 °C and 5% CO_2 with a CCM change every second to third day, respectively.

At a confluence of the cells of 80–90% sub cultivating was executed. PBS, Trypsin/EDTA, and fresh CCM were tempered prior to use. Cells were washed twice with PBS, incubated with 10ml Trypsin/EDTA for 5 min, to detach cells from the surface, followed by addition of 40ml CCM. Centrifugation at 1500 rpm, 3 min, is followed by resuspension in 10 mL CCM.

Cells were counted manually in a cell chamber described by Neumann and diluted afterwards as desired. Desired concentrations were 10^4 cells/mL for adhesion studies, 10^5 cells/ml for electrical measurements, and 10^6 cells /mL for sub culturing.
